# A Series of Triplets

**Published:** 1883-09

**Authors:** Henry Ogden

**Affiliations:** 243 N. Clark St.


### ARTICLE II.

A SERIES OF TRIPLETS. By HENRY OGDEN, M.D. Read before the Chicago Medical Society, June 18, 1883.

A TRIO OF TRIPLETS.—The occurrence of triplets being rare, and three cases occuring in this city recently within the space of seven weeks, I concluded it might be interesting to the medical profession to know something more in detail in regard to these cases.

By request of the editors of the JOURNAL AND EXAMINER, I have gathered up such facts as were attainable relating to them.

I first condense from Dr. Spiegelberg, the German author, a few general points in relation to membranes, ova., etc., in multiple births, after which I shall present the cases of triplets occurring here.

As high a number as five children at a birth have been known to occur, a preparation of which was seen by Dr. Spiegelberg.

I have not been able to find any authentic record of a higher number.

Some of the old French and German authors speak of some remarkable cases, but I believe none of them claim they actually saw these cases, but say "it was thus written."

The frequency of multiple births varies with the year and country.

Twins are said to occur once in eighty times, while triplets occur only once in 6,000 or 7,000 times, the number varying a little with different authors from the above.

Generally twins are of different sex, next in frequency are two boys, and least in frequency are two girls.

There are either two corpora lutea or one double one. Sometimes two come from one ovary and sometimes one from each ovary. This has been known only where post-mortems were had. The decidua is single except in cases of double uteri. The membranes may be double or single, as also the placenta. If the chorion is double there has been more than one egg.

Two separate placentæ may have grown together. If the eggs are far apart a decidua may separate them.

The placental circulations are separate if there are two chorions, or at least the anostomosis is very limited. If there is one chorion the fruit has come from one ovum. If there are three vesicles in one ovum and three placentæ, the placentæ are melted together and there is no trace of a septum between them. This was the condition in Dr. Bartlett's case.

In cases where there is only one ovum, a large anostomosis is necessary between the fœtal blood vessels, even between a vein and an artery. Twins in a common ovum are uniformly of the same sex, that is, they are alike, while children from different ova have such general resemblance only as offspring from the same parents commonly present.

In twins one single chorion occurs one time in eight, while the amnion is single only once in 132 times. In triplets and in other multiple cases, the same rule holds as to the placentæ and membranes, except, however, there are some complicated relations.

Commonly in triplets there exists one ovum alongside of a double one, yet there has been observed in triplets one chorion only, as also in cases of quadruplets. The relative frequency of a common amnion seems to increase with the number of children at a birth.

Generally in size and weight twins are below the average. But in twins at full term the aggregate weight is greater than in case of one child.

More commonly than in single labor is the fœtus discharged at the 39th to the 40th week, on account of the tension of the uterus. The mother's organs not being large enough for more than one, generally, besides the tension in the uterus, may be supposed to mechanically interfere with their development. Sometimes they are not developed alike, one small; one large. The latter may be supposed to have had better surroundings in the way of location for getting a blood supply from the uterus. This is often observed in double uteri cases. The size of the placenta, the length and thickness of the cord, corresponds to the size of the child.

The largest child is not always born first, as in Dr. Bartlett's case of triplets, the medium sized one first, the smallest next. and the largest child was born last. But in the other two cases now reported, the largest was first, the medium sized one next, and the smallest last.

CASE BY MIDWIFE.—MRS. M. LONDT.

The first case of triplets we note here occurred at No. 96 Fullerton avenue. Parents are German; were married in Germany, and one child born there. Moved to this city eleven years ago.

The father, Mr. Anton Arendt, is 40 years of age, is of medium height, but good weight. The mother, Julia Arendt, is 32 years of age, good size and weight, very fair complexion, and light blue eyes. She has given birth to nine children, three boys and six girls, including the triplets. At 3 o'clock, Sunday morning, February 11, 1883, the mother was awakened by some slight labor pains, but they were not very severe until after 6 o'clock, when the midwife was sent for. At about half past six o'clock her pains began to increase so that she was compelled to take to the bed.

The midwife did not arrive until 7:20 A. M., and at 7:30 A. M. the first baby was born. At 7:45 the second child came, and at 8 o'clock the third baby was born, there being only 15 minutes between the births of the first and second and second and third. All girls and living. At 8:10, or ten minutes after the last child was born, the placenta came away, being very large and apparently in one common mass, showing the three separate cells very distinctly, and the cords varying in length and size according to the size of the babies.

The first was a vertex presentation, while the last two were foot presentations. In this instance the largest child was born first, the medium sized one next, and the smallest one last.

The weights were 6½ pounds for the first, 5½ pounds for the second, and 4½ pounds for the third, a total of 16½ pounds. The babies are all in good health, although the youngest has been slightly ill at times. The mother nurses them, but as the amount of her milk is inadequate to the demands, the bottle is used to make up the supply.

There was a great amount of hæmorrhage, and the patient was in bed for about seven weeks, when she was able to be about the house.

She now complains of severe pains in joints, more in the right knee and foot. During term of pregnancy her legs became swollen occasionally, so that she was unable to stand on her feet for a part of a day at a time.

All of her previous labors were very much longer and more tedious than at this time. Mother and children were doing well at last accounts.*

CASE OF DR. H. VANBUREN, 15 N. MAY ST.

At a little after midnight on the morning of March 30, 1883, Dr. V. was called to the house of Mr. E. Garrison, 341 W. Randolph St., and found his wife, Alice Garrison, in labor pains, but not at all severe. She lingered along, with some inertia of the uterus, till 5:30 A. M., when she gave birth to the first boy. This was a breech presentation.

At 6 o'clock, the second boy was taken with the forceps, as there seemed to be a detachment of the placenta, cutting off the blood supply to the child. This was a vertex presentation. At 7 o'clock A.M., the third boy was still-born, and by appearances had been dead for several hours. This was a foot presentation.

These children were born within an hour and a half. In about

* The last born child died in June from inanition, having been weak from her birth.

20 minutes the placenta came away. There were separate and distinct cells and cords, and one common placenta. The cords were shorter than with single cases.

The first child weighed 5½ lbs., the second 5 lbs., and the third 4½ lbs., making an aggregate of 15 lbs.

The placenta was but little larger than in average single births.

There was only the usual amount of hæmorrhage, and the labor, on the whole, was not more severe, or the pains to exceed the average case of an easy single birth.

Patient had given birth to one child before, and she was now at full term. Patient's mother had twins.

Parents are American.

The father of these children is 35 years of age; tall and fleshy, weighing about 200 lbs. The mother is 25 years of age; she is about medium size and weight.

She was very well all through term and is getting on well now, and nurses the two surviving boys.*

CASE OF DR. JOHN BARTLETT, 450 N. CLARK ST.—TRIPLETS. FEMALE.

Parents are Swedish, residing at 1675 Gehrke avenue.

The father, Albin Nelson, 28 years of age, is tall and good size. The mother, Augusta Nelson, 24 years of age, weighs 135 lbs.; is blonde, of good stature and form. Was delivered of her first child by a midwife, January 26, 1881, twenty-six months prior to the birth of triplets.

Some weeks afterward, Dr. Bartlett was called, because of her slow getting up; found cellulitis existing, and also a want of normal involution.

These conditions yielded readily to the usual means, and on March 3, 1882, he was called upon to attend her in childbirth. The head was presenting favorably, but the labor was tedious from uterine inertia, so that it finally became necessary to deliver with the forceps. Her recovery was satisfactory.

On March 27, 1883, Dr. B. was again called to her, and she

---

* As noted above, the last born baby was still-born. The first born died July 9th. The second born died July 13th. Both were sick about one week with indigestion, and died of cholera infantum They were fed by the bottle after May 1st, but did n t do well.

was again in labor, and, as she thought, several weeks before her time.

Two weeks previously, she had greatly fatigued herself in walking, and a few hours afterward she was seized with violent neuralgic pains in the uterus, so that a miscarriage was feared. The patient called attention to her unusual size, and expressed jocosely a conviction that she would have twins; the uterus was very large. No indications of a plural pregnancy were detected upon palpation. The labor progressed normally, the pains being stronger and more efficient, and the os dilating more speedily than in the former labor. At 10 o'clock A. M., a child below the normal in size was born, the presentation being cephalic.

Struck by the relative smallness of the child and the yet large size of the uterus, apparent at a glance, the cord was tied as promptly as was proper, and attention given to the mother.

Pains were recurring, and an additional sac of membranes was gathering in the os. Through these it could be determined that neither extremity of the fœtal ovoid was presenting, and accordingly he ruptured the membranes at once, and sought to discover the position of the child. The right side of the abdomen presenting, the feet were at once sought, and the child turned and promptly delivered. The second child was considerably smaller than the first. This being removed, the womb was noted to be larger than after complete delivery, and a third sac was found descending into the os. Discovering here, likewise, through the membranes, that a cross-birth existed, he ruptured the membranes, and found a third child presenting, with right humerus and thorax toward the os. The child was promptly turned. It descended readily till the head engaged in the brim, gentle traction having been made upon it. Finding both arms extended alongside of the head, the ordinary rules were disregarded, and the head pulled through with the arms as mentioned. This traction was such as to approach force, but not violence.

The first and second child breathed promptly; this last was slow to respire, so that the ordinary restorative measures, including artificial respiration, were used, but in a few minutes breathing was established and the child removed. This completed the birth of three girls.

Notwithstanding the rather speedy emptying of the uterus, there being only five minutes intervals between the birth of the children, the womb responded quickly and well to the manipulation of the hand through the abdominal walls, and promptly contracted on the placenta.

It was noticed that it was in this stage unusually large. Gentle pressure and friction was maintained for some time. There was no hæmorrhage, nor could the placenta be felt by the finger per vaginam. After the lapse of fifteen minutes, a gush of blood occurred, apparently about 7 or 8 ounces. No sooner had this been removed than a second gush followed, as copious and sudden as the first. It was evident that the placenta was partly detached and a violent post partum hæmhorrhage was probable, if energetic measures were not at once adopted. The hand was passed into the uterus and insinuated between the membranes and its walls. The edge of the very large placenta was reached at a point not separated from the uterus. Detachment was here begun and carefully continued over the whole attached surface. Contraction ensued and no more than ordinary hæmorrhage followed.

This placenta was single, weighing 2 pounds 6 ounces and measuring 9¼ by 9½ inches across. The largest cord measured 18½ inches in length, the medium 17 inches, the smallest 14½ inches. The amnions were separate and the cells were proportionate in size to the cords and weight of the children, which were as follows: 6 pounds one ounce for the first, 4 pounds one ounce for the second, and 6 pounds nine ounces for the last born, aggregating 16 pounds 11 ounces. The patient did perfectly well till the 10th day, when over-exertion in the care of the children brought on a threatening of cellulitis, which, however, was apparently aborted within a few days. But from exposure and imprudence in exercise, a decided attack of cellulitis occurred on the 24th day, which confined her to the bed for three weeks.

The inflammatory exudation, as large as a child's head, was confined to a part of the true and false pelvis on a level with, to the left of, and behind the uterus. Recovery was finally complete. In connection with this attack of cellulitis it is well to state some additional facts. It will be noticed that in the delivery of the third child, Dr. B. followed the precepts of Deventer, and withdrew the head with the arms extended alongside of it. In so doing, he was careful to place the diameter of the child presented by the head plus the thickness of the arms in a long diameter of the superior strait, the left oblique. It has been stated that in the extracting this head a degree of traction was used more than is commonly found necessary in delivering the after coming head in multiparæ.

It was noted that more than usual pain was complained of under these manipulations; possibly the cellulitis which followed may have been in part due to bruising of her tissues by the passage of the enlarged diameter presented by the head and extended arms. As there are those who may question the practice of leaving the arms extended in this case, it may be well here to state that it was not with the object of experiment that Deventer's plan was adopted in this instance, but in part from the urgency of the case. The arms were above the head at the superior strait; pulsation had ceased in the cord; promptness in delivery was demanded, and in this extremity, Dr. B. recalled and relied upon the precepts of Deventer. As all present may not be familiar with the instructions of the old authority referred to, it may be well to state them in brief.

In his first edition, 1724, he says:

"Whereas all the authors that I know teach to the contrary, I positively advise that (in delivering by turning) the arms be left (extended) about the head, to be excluded along with it. In my practice, everything has succeeded very well by this method, not so much as one head having stuck in the mouth of the womb.

"If the head passes with difficulty, which seldom happens, you may draw down one hand or the other, but both hands are never to be drawn down; that would do more mischief than good.

"In turning in the early stage of labor, neither the mother nor the child are exposed to the danger of death.

"The whole matter (success in turning) depends upon two things:

"First, the proper introduction of the hand. Second, the greatest care should be taken that the head and arms pass together."

These citations from Deventer formed the basis of remarks made by Dr. Bartlett before the Gynæcological Society, in December last, in a paper entitled "A Lesson from Deventer."

In the case before us, the diameter formed by the head and arms of the child pressed against the temples, measured 4¼ inches, exactly the same as the transverse diameter of the inferior strait, a relation demanding, even in this undersized child, a compression of soft parts. In conclusion, I will quote some figures from Scanzoni, the well-known German author, relating to the frequency of multiple births.

From a table taken from the registration of births in Prussia, between the years 1826 and 1849, a period of 23 years, it would appear that there were 13,360,575 births. Of these, there were 149,964 cases of twins, 1,689 cases of triplets, and 36 cases of quadruplets. In other words, there were twins once in 89 cases, triplets once in 7,910 cases, quadruplets once in 371,126 cases, and no case of a higher number appeared upon the record.

AUGUST 1, 1883.

After reading the "Trio of Triplets" paper to the Chicago Medical Society, other cases of triplets occurred in this city, and as there were some points of interest in them, I subjoin a brief description of these cases, also, some points in a case of triplets occurring at Mendota, Ills., two years ago.

CASE ATTENDED BY DR. J. TASCHER, corner Ashland and Chicago Avenues.

Parents German, residence, 82 Emma street; married 13 years. Father August Vogt, 41 years of age, a little under the medium size, and by occupation a blacksmith. Mother Frega Vogt, 32 years of age; good size and stature, blonde, with blue eyes. She has given birth to nine children, two boys and seven girls, including the triplets. No history of multiple births in the family.

On the morning of June 20, at about 6 o'clock, pains came on and Dr. T. was called to her. On examination the membranes were found to be ruptured, shoulder presentation, with one hand in vagina. At the side of the shoulder was felt what seemed to be one or more hands covered by an unruptured bag of waters. This in connection with the excessively large uterus, led to the conclusion that twins were forthcoming. It was thought best to administer chloroform, but an examination of the heart revealed a mitral deficiency, so the administration of chloroform was dispensed with for evident reasons. Podalic version was performed with but little pain to the patient. Almost immediately after the feet were brought down No. one was born.

On examination found a second bag of waters and a hand presenting, ruptured the membranes and turned No. two without difficulty, after which it was born without further assistance.

The uterus being still quite large and pains energetic, examination was again made, and this time a vertex presentation was found, with membranes intact. These were ruptured and No. three was born without further delay.

After this a drachm of fld. ext. ergot was administered and used Crede's method to insure uterine contraction. In a short i me three separate placentæ were expelled. But little hæmorrhage followed, barely enough to soil the clothing. Labor was completed in about an hour after the first child was turned. The three female children were born before 8 o'clock A. M.

The babies weighed respectively at birth 5 lbs, $5\frac{1}{2}$ lbs, and 5 lbs, aggregating $15\frac{1}{2}$ lbs.

Mother kept her bed nine days, when she was about the house. Says she was about two weeks short of full term. Had swelled feet two weeks before the babies were born, but otherwise was quite well, thinks this very much the easiest labor she ever had.

CASE BY DR. P. J. ROWAN, 138 S. Desplaines street.

Parents are Irish-American born.

Residence, 608 Dickson street.

Father, James Quigley, is 30 years of age, medium size, good stature, weighs about 180 pounds.

Mother, Kate Quigley, is 27 years of age, rather small in size and stature, not weighing over 100 pounds; black eyes and dark complexion.

Has given birth to three children before the triplets; one boy and two girls, and now three girls.

Pains began at 1 o'clock P. M., July 8, 1883, and Dr. R. was sent for. Upon arrival it was found that the pains were light, but had been quite effectual, as the os was well dilated. The membrane was ruptured, and at 5 P. M., the first female child was born without difficulty. Another membrane engaged in the os was ruptured, and at 5:15 P. M. the second girl was born as easily as the first. These two were foot presentation. At 5:30 the third girl baby was born, this presentations being cephalic. A drachm of ext. ergot fl. was administered, and in a few minutes the placenta came. It was in two parts; one was by itself, except being connected to the other mass by a small, thin sheet of membrane, which easily gave way and was not vascular. The other two were in one mass, with fissures or notches on each side, midway. But there is perfect anastomosis, as the two are melted together.

The chorions and amnions were separated, and each sac seemed to contain as much liquor amnii as is found in ordinary single cases, thus giving the mother a remarkably disproportionate size. The uterus contracted well, so that there was only slight hæmorrhage.

At the birth of her first child, some years ago, there was a laceration of the perineum reaching quite to the sphincter ani muscles, which still exists. This may account for the easy expulsion at this time, while forceps have been used in the previous births. Patient has made a good recovery, being in bed about ten days. The first baby weighed 4¼ pounds, the second 3¾ pounds, the third 3¼ pounds, a total of 11¼ pounds. The third born child died three days after birth, the second five days after birth, both from inanition. The first born lived twenty-three days and died of convulsions.

CASE OF DR. J. W. EDWARDS, MENDOTA, ILL.

Parents Irish.

Father, Mr. C., is Irish-American born; 36 years of age; 5 feet 10 inches in height, and weighs 180 pounds.

Mother, Mary C.; native of Ireland; 35 years of age; medium stature, weighs 150 pounds, is mother of eleven children, having had four boys and four girls before the triplet girls, this being her ninth labor.

At 2 o'clock A. M., June 3, 1881, patient was awakened by pains which increased in frequency and force, and at 3 o'clock Dr. E. was called.

The membranes were found to be ruptured, and the child's head engaged at the superior strait. This was a vertex presentation.

A few severe pains completed the delivery of a female child at 3:30 A. M.

Finding the uterus still larger than at full term, and evidence by motion of the presence of a second child, using gentle circular friction, pains came on and membranes engaged in the os. They were ruptured and vertex presentation determined. After a few pains a second girl was delivered at 4:20 A. M.

It was found that the womb was still as large as in a case at full term, and a third examination detected another set of membranes presenting. These were ruptured as before, and after three pains the third female child was delivered at 5:20 A. M. This was a vertex presentation.

The uterus contracted down well upon the expulsion of the third child, and in about fifteen minutes placenta No. one was thrown into the vagina and removed. Immediately following was Nos. two and three, partially attached to each other by membranes, all presenting a separate cell and cord, each placenta was a little more than half the usual size of the average single case.

The weights of children were for No. one 6 lbs, No. two 7½ lbs, and No. three 7 lbs, aggregating 20½ lbs.

There was only the usual hæmorrhage, and the labor shorter than any of the eight preceding, as the children were always large, averaging ten pounds, and the first and second stages of labor were never less than the whole time required in the delivery of these three children. Patient was, however, slower getting up at this time, but recovery was complete. No history of twins or triplets in the family. These triplets are now two years old and well.

Condensing from these six cases, we find the nationality to be two German, one Swede, two Irish and one American.

Five of these cases were females, while one case was males. There is no history of multiplc births in families, except one patient's mother had twins.

In each case the labor was much easier and shorter than any preceding, and in no case was the hæmorrhage up to the usual amount. In three cases the placenta was common, with, however, separate cells, and in three cases the placentæ were separate.

In the six cases, there were nine foot presentations, eight vortex and one breech presentation.

Each patient has made a complete recovery.

Dr. L. H. Montgomery has reported to me a case of triplets, of German parents, in the summer of 1869, near Shullsbury, Wisconsin.

The case was a farmer's wife, some five miles from the town, and Dr. M. saw the case with his preceptor, and they were able to report everything favorable, and that none of them had gotten away, etc. When the second child was born, the mother fainted, and remained unconscious during the birth of the third child (only a short interval, however), and she was not aware of the birth of a third child until told of it, when she exclaimed in her teutonic dialect: "Das ist genug." The children possess unusual intelligence, and are foremost in their studies. The surnames of the two boys are John William and William John, the girl, Sarah. There are such strong resemblances in the boys' facial features that their most intimate acquaintances have to turn them around to recognize them, which is often done by school mates. They are all quite well, and, as above stated, are unusually bright.

243 N. CLARK ST.

---

DRS. DE LASKIE MILLER and Wm. H. Bradley, of Chicago, are in Europe. Will return in September.

DR. R. L. LEONARD, of Chicago, is visiting on the Pacific Slope.

